# Asylum Officials and Their Pensions

**Published:** 1897-08-28

**Authors:** 


### Asylum Officials and their Pensions.

Taken as a whole our county asylums are institutions of which the nation may well feel proud, however much the necessity for their existence is to be deplored. But they have only maintained their high place by dint of the constant efforts of their permanent officials.

With the Lunacy Act of 1890 an amount of unnecessary work was thrown on the officers that would have damped the energies of almost any other men; but their love of the cause and of the system in which they had been bred sustained them, and while the medical spirit could not fail to be sadly bruised by the Act, it was a matter of no small gratulation that it was not entirely crushed. Another Lunacy Act Amendment Bill was lately be-before the House of Lords, of which, in a certain sense, the most important clause, and the only one we shall touch on at present, was that relating to the pensions of asylum officials. At present the pensions clause is a permissive one only; but it has been almost invariably acted on, and an officer or official of any kind who had served fifteen years and was fifty years of age felt sure of being justly treated.

The Bill proposed the adoption of the Poor Law Officers' Superannuation Scheme, which requires forty years' service and a minimum age of sixty. It is manifest that such a scheme is not applicable to our county asylums. Only the very strongest and most robust of men and women could stand the constant strain of being in attendance on the insane for forty years, and if it were possible to hold out so long, the individual would be useless to the institution. Fancy the wards of an asylum where any considerable proportion of the attendants or nurses was between fifty and sixty years of age! The very idea provokes a smile, and were it ever to become an established fact the inevitable result would be that deputies and assistants would be called for from all quarters, and the salaries of these deputies would exceed the pensions which the others ought to have had, so that from an economical standpoint the proposal would be a mistake.

The Visiting Committee of one county asylum has undertaken to suggest that the Lord Chancellor should insert the following sub-section: "Any officer who is in constant attendance, medical or otherwise, on patients in the wards of a lunatic asylum, and who in such capacity has completed an aggregate of twenty-five years' service, shall have the option of retiring after the age of fifty with a pension calculated upon the basis set forth in the said Poor Law Officers' Superannuation Act."

We should consider this a satisfactory solution of the question, and we hope it will be loyally supported by the Visiting Committees of other asylums, and inserted in the Bill, which will make its appearance again next session. It is clear that some important modification of the clause is imperative, especially if the Act is intended to be retrospective. Officers have been induced to enter and remain in asylum service to a great extent by the hope or certainty of a moderate pension after a reasonable period of service, and any great departure from these conditions would be so like a breach of faith that we feel sure it only needs to be pointed out to be attended to. It scarcely meets the case to say that officials may remain under the old Act if they please; because with a compulsory pensions clause in one Act and a permissive one in another, somebody would be sure to suffer. Moreover, the passing of such a clause as the one in the Bill would almost certainly lead to a speedy deterioration of the raw material from which asylum officers and attendants are made, and thus the insane themselves would soon begin to suffer.

